# Systemic Nanoparticle‐Mediated Delivery of Pantetheinase Vanin‐1 Regulates Lipolysis and Adiposity in Abdominal White Adipose Tissue

**DOI:** 10.1002/advs.202101789

**Published:** 2021-06-24

**Authors:** Siyu Chen, Wenxiang Zhang, Chen Sun, Mingming Song, Shuang Liu, Mengyi Xu, Xiaojin Zhang, Li Liu, Chang Liu

*Adv. Sci*. **2020**, *7*, 2000542

DOI: 10.1002/advs.202000542

In the originally published article, one affiliation of Chang Liu was missing. Please find the complete affiliations here:

Dr. S. Chen, Dr. W. Zhang, C. Sun, M. Song, S. Liu, M. Xu, Prof. C. Liu

State Key Laboratory of Natural Medicines, School of Life Science and Technology

China Pharmaceutical University

Nanjing 211198, China

Dr. X. Zhang, Prof. L. Liu

Department of Geriatrics

First Affiliated Hospital with Nanjing Medical University

Nanjing 210029, China

Prof. C. Liu

State key Laboratory of Pharmaceutical Biotechnology

Nanjing University

Nanjing 210046, China

E‐mail: changliu@cpu.edu.cn

The authors apologize for any inconvenience this may have caused.

